# Analysis of urban subway construction collapse disaster chain and research on chain breaking and disaster reduction

**DOI:** 10.1371/journal.pone.0318269

**Published:** 2025-03-26

**Authors:** Ying Zhang, Yingying Gu, Ningning Lian, Lei Peng, Yu Hao, Wei Wang, Rumeng Tian

**Affiliations:** 1 School of Emergency Management, Henan Polytechnic University, Jiaozuo, Henan, China; 2 Safety and Emergency Management Research Center, Henan Polytechnic University, Jiaozuo, Henan, China; 3 Henan Polytechnic University, Jiaozuo, Henan, China; 4 School of Energy Science and Engineering, Henan Polytechnic University, Jiaozuo, Henan, China; Southwest Petroleum University, CHINA

## Abstract

In recent years, China ‘s major cities set off a climax of subway construction, but also brought an endless stream of safety accidents. In order to analyze the impact of the evolution process of urban subway construction collapse disaster on residents ‘ life and social economy, by collecting typical cases of subway construction collapse disaster, combined with disaster chain and complex network theory, the network model of subway construction collapse disaster chain is constructed, and the key node events and key propagation paths are analyzed. Based on this, targeted chain-breaking disaster reduction measures are proposed. The results show: the collapse disaster chain of urban subway construction can be divided into early, middle and late stages of disaster evolution. Through the destruction of collapse, underground pipeline rupture, road damage, affecting the lives of residents and building damage and other key nodes or cut off the collapse →  underground pipeline rupture, road damage →  traffic paralysis, collapse →  building damage, construction technology is not standardized →  collapse, construction equipment failure →  collapse and other key effects are significant. The relevant research results can provide a knowledge map for effectively coping with the collapse disaster chain of urban subway construction, identify key nodes and propagation paths, and establish strategies for emergency response and chain-breaking disaster reduction.

## 1. Introduction

In China, with the rapid development of urbanization, urban rail transit has effectively alleviated the traffic congestion problem in big cities with its advantages of large scale, fast speed, favorable price, comfortable and convenient, and good environmental benefits [1]. According to the annual data of China Urban Rail Transit Association 2023, the scale of urban rail transit operating lines in the Mainland continues to expand, with a total length of 11,224.54 kilometers [2]. However, most of the subway construction projects are located in urban areas, with dense population and dense buildings. If disasters occur at the construction site, it will be very difficult for construction personnel to shelter, emergency evacuation and later rescue;Moreover, the subway construction environment is in a complex underground space, and various materials, machinery, pipelines and lines can easily lead to derivative accidents. For example, the worst subway collapse in China’s history, the 2008 Hangzhou subway collapse, caused 21 deaths and 24 injuries, severely affected the lives of nearby residents, and caused direct economic losses of RMB 49.61 million [3].

Disaster chain refers to a series of disaster phenomena with causal relationship formed by triggering or inducing one or more other disasters to occur one after another [4–5]. Its definition focuses on the causal relationship between primary disasters and secondary disasters [6], showing the characteristics of mutual influence and close connection between disaster events [7], which is characterized by the chain reaction between disaster events in the space-time dimension and the accumulation and amplification effect of losses [8]. At present, the research on disaster chain mainly focuses on the aspects of disaster system and evolution law, and explores the weak links in disaster chain such as geology [9], meteorology [10], earthquake [11], fire [12], flood [13], etc., so as to provide reference for formulating disaster prevention and mitigation measures. In the field of subway, scholars have carried out more research on disaster chain in the operation stage. Taking the flooding events of subway line 5 and Beijing-Guangzhou expressway tunnel caused by Zhengzhou ‘7·20’ rainstorm as an example, Wang Hao constructed a knowledge map to analyze the transmission mechanism of disaster chain, and identified the key links of disaster chain, including induction point, trigger point, diffusion point and amplification point, so as to enhance the timeliness of urban flood warning and the effectiveness of response [14]. Jiao Liudan used Jaccard index and Bayesian theory to analyze the evolution probability of urban rail transit disaster chain. In the process of rail transit operation, the probability of operation interruption and casualties caused by equipment failure is the highest [15].

However, most of the current research focuses on the impact of metro operation process, such as flood, fire and equipment failure on the system, and lacks the whole process analysis of the collapse disaster chain during metro construction, and the analysis of its evolution mechanism is not deep enough. According to the statistical data of SCAD [16] database, more than 30% of the accident cases are collapse accidents. Collapse accidents are more dangerous than other types of accidents, and often cause higher death and serious injury rates in subway construction sites [17]. The collapse of subway construction in Chinese cities not only causes huge losses due to the dense population, economy, infrastructure and other disaster-bearing bodies, but also easily induces a series of disaster events due to the complex urban system and high correlation, forming a complex subway construction collapse disaster chain [18]. Therefore, by collecting typical cases of subway construction collapse disaster, this paper summarizes the elements of disaster system and establishes a complex network of induced transmission relationship, identifies key node events and propagation paths, and analyzes disaster reduction measures through different index results. This has reference value for scientific understanding of the collapse disaster chain of subway construction, and provides a scientific basis for comprehensive disaster reduction, disaster preparedness and disaster relief of urban subway construction collapse disasters.

## 2. Metro construction collapse disaster system analysis

The disaster system is composed of disaster-inducing factors, disaster environment and disaster-bearing bodies, and disaster risk is a product of the interaction of the subsystems. The environment in which disasters occur is the basis for their conception, birth, development and evolution, and is characterized by a certain degree of sensitivity and variability. Disaster-inducing factors are factors that have a hazardous nature in terms of loss and adverse effects on life, property, and the environment. The disaster bearing body refers to the object of injury and damage, which is vulnerable

### 2.1. Disaster environment

(1) The natural environment includes hydrology, soil, meteorology and topography. Most of the subway construction projects are underneath heavily traveled roads, and it is difficult to accurately predict the geological and hydrological conditions prior to construction, so there are many potential safety hazards [19]. The occurrence of meteorological disasters will lead to various secondary and derivative disasters, such as continuous rainfall or heavy rain in summer, which can easily lead to accidents such as foundation pit instability and collapse. The geological features of the city are related to the occurrence of accidents. For example, the soil in Shenzhen and Guangzhou is unstable, and the soil is soft and prone to collapse. If the geological exploration is incomplete, it is easy to lead to low construction quality and control measures are not applicable [20].
(2) Urban underground space buried a large number of electrical and plumbing facilities make the construction environment more complex, if an accident occurs at the construction site, not only very easy to cause mass deaths and injuries and property damage, but also affect the daily life of the surrounding residents caused by water, electricity and traffic congestion, resulting in a public event to bring about a bad social impact. And in urban areas, due to the influence of surrounding buildings, pipelines, etc., in order not to affect the normal life of residents, the construction scope is small, making the construction work environment poor.

### 2.2. Disaster-inducing factors

According to the cases of subway construction collapse accidents, while referring to related scholars’ literature, the disaster-causing factors leading to subway collapse accidents can be summarized, and the disaster-causing factors are divided into four major categories.

(1) Poor construction environment. Poor environment accounts for a large proportion of the disaster-causing factors, mainly around the construction site environment is poor, including the natural environment is poor, the surrounding environment is poor, the construction site environment is poor in three aspects. Due to the insufficiently comprehensive inspection of the surrounding buildings and underground pipelines, and the lack of effective monitoring of the groundwater level, this has resulted in a poor surrounding environment and poor natural conditions during construction, thus making the construction site worse. Poor natural conditions such as poor geology, persistent rainfall, presence of ground cracks, etc. The construction site environment is affected by the adverse effects of equipment, the behavior of personnel, the condition of materials, and the day-to-day management of the site.
(2) Construction techniques are not standardized. In the early investigation stage, the designer ‘s inadequate geological survey leads to the error between the construction plan and the actual situation, which affects the subsequent construction and leads to the collapse. Secondly, the construction program is not standardized or the workers do not operate in violation of strict compliance with the standards of construction processes, techniques and other standards and guiding documents such as the construction program.
(3) Man-made management deficiencies. Most accidents occur from the unsafe behavior of people and the unsafe state of things, in construction accidents management factors and the unsafe behavior of workers is the main cause of accidents, so good management is the key to [21]. In the construction process some construction units will be regulations in form, safety awareness is weak, in the organization and management do not perform safety management duties, safety production training is insufficient, the main responsibility for safety production is not implemented. Improper emergency response measures, unauthorized command, and incomplete investigation of hidden dangers in construction management lead to problems in the construction process, resulting in serious consequences of the collapse.
(4) Construction equipment failure. The generation of failures in equipment and facilities is influenced by the supervision of safety inspections, human capacity and geological conditions. If the daily safety production inspection and supervision becomes a mere formality, there is no inspection and supervision of the mechanical equipment and whether it is correctly operated and used, there are defects in the daily on-site management, and the safety awareness is poor. Failure to maintain and repair the equipment in time will lead to mechanical equipment failure. Insufficient technical experience of workers, failure to use equipment correctly, irregular operation, etc. Secondly, poor geology may make large machinery and equipment unsupportable, triggering equipment destabilization. All of these can create unsafe behaviors and lead to defective construction equipment.

### 2.3. Disaster bearing body

**Fig 1:**
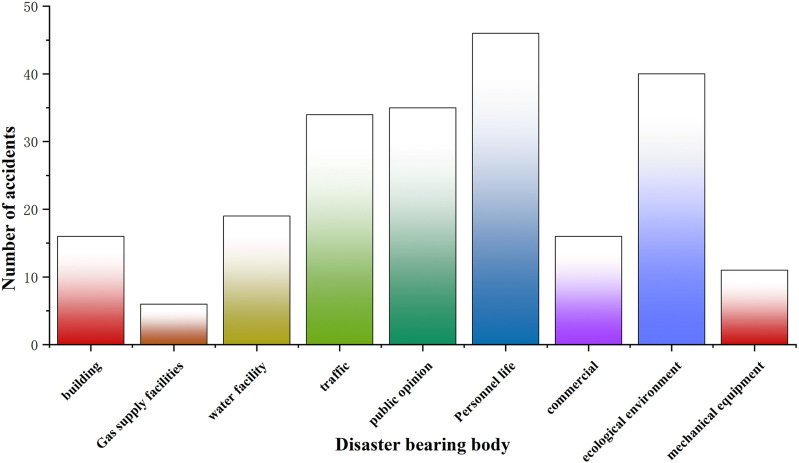
Frequency distribution of disaster-bearing body of collapse disaster in urban subway construction.

The disaster-bearing bodies of urban subway construction collapse disasters include human lives, buildings, transportation, water supply facilities, gas supply facilities, social opinion, ecological environment, machinery and equipment, and commerce. Through the statistics of the frequency of damage to the disaster-bearing body in the collapse accident of subway construction (Fig 1). It can be seen that most of the collapse accidents show a chain reaction situation, endangering people ‘s lives, causing serious casualties and economic losses. Complex underground pipelines, once the collapse is also very easy to cause gas leaks and water pipe bursts, resulting in water and gas cuts affecting the normal life of residents. When the ground collapses, the site is damaged, generating dust, fumes and other harmful gases and toxic substances, and pollutants spread to the atmosphere and groundwater affecting the ecological environment.

## 3. Subway construction collapse disaster chain construction

This paper collects 145 cases of subway construction collapse accidents that have aroused widespread concern in the society from 2000 to 2023, mainly relying on authoritative channels such as the Ministry of Emergency Management of the People ‘s Republic of China, the official websites of local governments, the websites of the Ministry of Housing and Urban-Rural Development and the reports of online media. The accident cases were collected by keywords such as ‘ subway ‘, ‘ subway construction ‘, ‘ subway accident ‘ and ‘ subway construction safety accident ‘.

**Fig 2:**
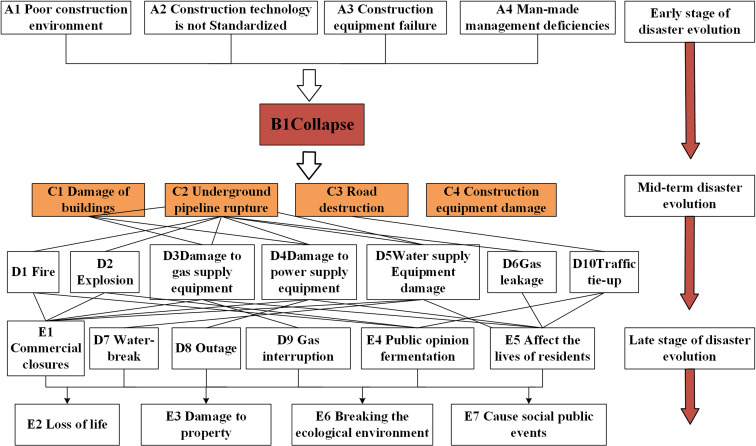
Urban subway construction collapse disaster chain.

The collapse disaster evolution process of subway construction is divided into three parts: the early stage of disaster evolution, the middle stage of disaster evolution and the late stage of disaster evolution. The collapse disaster chain of urban subway construction is constructed as shown in (Fig 2). The early stage of disaster evolution refers to the induction of primary disasters in a specific environment under the influence of disaster-causing factors. According to the statistical sample data of subway construction collapse accident cases, and referring to the relevant scholars ‘literature [22], the disaster-causing factors leading to subway construction collapse can be summarized, and the disaster-causing factors can be divided into poor construction environment, non-standard construction technology, human management defects, and construction equipment failures. The primary disaster triggered by the disaster-causing factors in the disaster chain is the collapse of subway construction.

In the middle stage of disaster evolution, primary disasters can rapidly trigger secondary disasters. The scope of this paper’s delineation of secondary hazards is based on the impacts that occur within a short period of time after the disaster. Urban subway construction collapse disaster secondary disasters mainly include building damage, underground pipeline rupture, road damage, construction equipment damage and subway tunnel body damage, which in turn triggered fires and explosions, etc., and the next secondary disaster will trigger new disasters, with a chain reaction posture.

Derivative hazards develop later in the evolution of a disaster and are indirectly triggered by secondary hazards. In this paper, derivative disasters are limited to losses that occur long after the disaster has occurred, such as commercial closures, public opinion, impacts on residents’ lives, and transportation paralysis, etc., and finally derivative disasters develop into public social events, which simultaneously trigger casualties and economic losses, and have a great impact on the ecological environment.

## 4. Risk analysis of subway construction collapse disaster chain based on complex network

### 4.1. Construction of complex networks

A complex network is a complex network topology with a large number of nodes and edges. Currently, many systems can be regarded as complex networks, such as social networks [23], rail transportation networks [24], and disaster chain networks [25], and complex network theory can be used to study the commonalities between different complex networks [26]. There are many disaster factors in the collapse disaster chain of urban subway construction, and the connecting edges between disaster factors are crisscrossed with obvious complex network characteristics. At present, the main tools used for complex network analysis include Pajek, Network X and Gephi software. In this paper, Gephi software is used. The software has an intuitive and easy-to-operate user interface. It is suitable for analyzing and processing various complex networks and systems, especially in network visualization graphical analysis. By analyzing the disaster chain with the help of complex network theory, the attribute characteristics of nodes and edges in the collapse disaster chain of subway construction can be grasped more deeply and conveniently, so as to provide reference for preventing the evolution of disasters.

The node numbering of the disaster elements in the constructed urban subway construction collapse disaster chain (Fig 2) is shown in (Table 1). Among them: A represents the disaster-causing factor; B represents the primary disaster; C and D represent secondary disasters; E represents derivative disasters, a total of 27 key nodes.

**Table 1:** Node numbers in the complex network of urban subway construction collapse disaster.

Numbering	Node name	Numbering	Node name
A1	Poor construction environment	D5	Water supply Equipment damage
A2	Construction technology is not Standardized	D6	Gas leakage
A3	Construction equipment failure	D7	Water-break
A4	Man-made management deficiencies	D8	Outage
B1	Collapse	D9	Gas interruption
C1	Damage of buildings	D10	Traffic tie-up
C2	Underground pipeline rupture	E1	Commercial closures
C3	Road destruction	E2	Loss of life
C4	Construction equipment damage	E3	Damage to property
C5	Damage to the main body of the tunnel	E4	Public opinion fermentation
D1	Fire	E5	Affect the lives of residents
D2	Explosion	E6	Breaking the ecological environment
D3	Damage to gas supply equipment	E7	Cause social public events
D4	Damage to power supply equipment		

Based on the analysis of the urban subway construction collapse hazard system, such as the proportion of primary hazards, the proportion of disaster-causing factors leading to subway construction collapse, and the frequency of disaster damage to the disaster-bearing body, the edges between the two nodes are assigned values. The specific assignments refer to the weight assignment criteria in reference [27], {1, 3, 5, 7, 9, 11} against {impossible, extremely difficult, not easy, probable, highly probable, certain}, respectively, which represent the difficulty of risk evolution of the former node to the latter node. A total of 66 key edges are extracted from the constructed urban subway construction collapse disaster chain, and the weights are assigned to the edges in the complex network of urban subway construction collapse disaster, as shown in (Table 2).

**Table 2:** Weight assignment of edges in the complex network of urban subway construction collapse disaster.

Start→Finish	weight value	Start→Finish	weight value	Start→Finish	weight value
A1 → B1	7	C2 → D5	5	D3 → D9	9
A2 → B1	9	C2 → D6	11	D3 → D6	9
A3 → B1	7	C3 → C2	5	D4 → D8	9
A4 → B1	9	C3 → D10	9	D5 → D7	9
B1 → C1	9	C3 → E2	9	D6 → D1	9
B1 → C2	5	C4 → C3	9	D6 → D2	5
B1 → C3	11	C4 → E3	11	D7 → E1	7
B1 → C4	9	C4 → E2	9	D7 → E5	7
B1 → C5	3	C4 → E6	9	D8 → E1	7
C1 → D3	9	C5 → E2	9	D8 → E5	7
C1 → D4	9	C5 → E3	11	D9 → E1	7
C1 → D5	9	C5 → C4	9	D9 → E5	7
C1 → C2	7	C5 → E6	9	D10 → E5	7
C1 → C3	7	D1 → D2	5	E1 → E5	7
C1 → C4	7	D1 → E2	5	E1 → E3	9
C1 → E2	9	D1 → E3	9	E2 → E3	11
C1 → E3	11	D1 → E6	7	E2 → E7	5
C2 → D1	7	D1 → E1	5	E4 → E7	7
C2 → D2	5	D2 → E1	9	E5 → E4	3
C2 → D3	9	D2 → E2	9	E7 → E2	3
C2 → D4	9	D2 → E3	9	E7 → E3	3
C3 → E6	9	D2 → E6	5	C1 → E6	5

**Fig 3:**
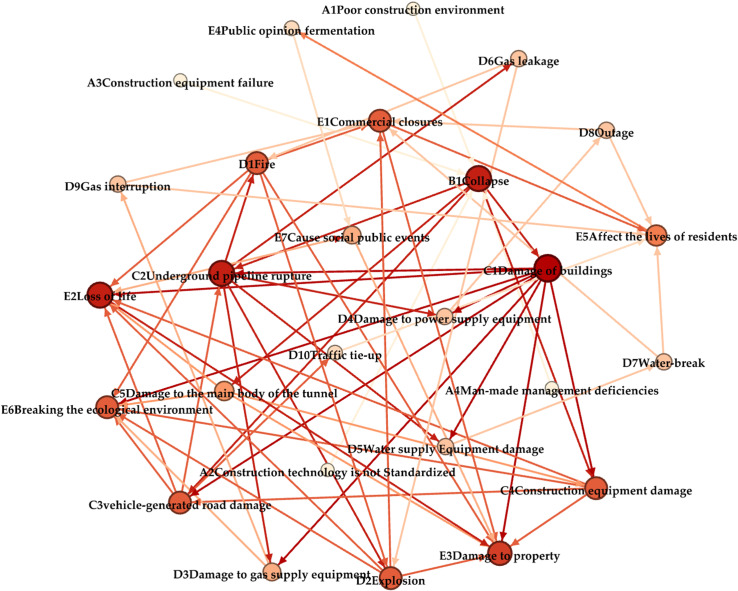
Visual topological structure diagram of complex network of urban subway construction collapse disaster.

In this paper, Gephi software is used to construct a complex network model of urban subway construction collapse disaster. Firstly, the numbered nodes and weighted edge data are imported into Gephi software. Subsequently, the parameters of the visualization page are optimized and adjusted to present a visual topology of the complex network of urban subway construction collapse disasters (Fig 3). The diagram intuitively reveals the relationship between the elements in the collapse disaster chain of urban subway construction. The darker the color and the larger the size of the nodes in the figure, the higher the in-out degree is. The direction of the connection between nodes represents the path of disaster development. The thickness of the connection indicates the difficulty of evolution. The thicker the line, the easier the evolution process.

### 4.2. Identifying disaster critical node events based on node metrics

By calculating the degree, closeness centrality and betweenness centrality of nodes, the importance of disaster event nodes in the collapse network of urban subway construction is analyzed.

(1) Degree

As a basic index to evaluate the importance of network nodes, the degree centrality of nodes is positively correlated with the importance of nodes [28]. The centrality of the node is the sum of the out-degree and the in-degree. The higher the degree, the more serious the consequences caused by this node in the network. The formula is:


$$D_{ci}=\overset{n}{\underset{j=1,j\neq i}{\sum }}\sigma_{ij}$$ (1)

In the formula:$D_{ci}$ is the node degree centrality, $\sigma_{ij}$ represents the number of edges between nodes and nodes, n is the total number of nodes in the network. In the chain evolution network of subway construction collapse disaster constructed in this paper, the degree centrality of C1 (building damage), B1 (collapse) and C2 (underground pipeline rupture) is ranked in the top three. These crisis events constitute important nodes in the evolution process of disaster situation, which should be paid special attention to in the safety prevention and control measures of subway construction.

(2) Closeness centrality

The closeness centrality of a node refers to the location of a node in the network, which reflects the average distance between the node and other nodes in the network. The higher the closeness centrality of a node is, the more critical the position of the node in the network is [29].


$$C_{ci}=\frac{n-1}{\sum_{j=1}^{n}d_{ij}}$$ (2)

Where: $C_{ci}$ denotes the closeness centrality of a node,$d_{ij}$ represents the number of edges in the shortest path starting from node *i* and ending at node *j*. The closeness centrality of E2 (casualties), E7 (social public events) and E4 (public opinion fermentation) events is the largest, which is the key to system risk management and control.

(3) Betweenness centrality

Betweenness centrality is to measure the number of shortest paths passing through a node in the shortest path connecting all node pairs in the network. The higher the betweenness centrality of the node is, the more significant the control or influence exerted by the node on the network is [30].


$$\begin{array}{l} B_{ci}=\sum \begin{array}{l} s\neq t\neq v,s,t\in v \end{array}\frac{\sigma \left(s,t\text{|}v\right)}{\sigma \left(s,t\right)} \end{array}$$ (3)

In the formula: $B_{ci}$ represents the node betweenness centrality,$\sigma \left(s,t\right)$ denotes the cumulative number of all shortest paths between node *s* and nodet,$\sigma \left(s,t|v\right)$ represents the number of shortest paths through nodev in the path from node *s* to node *t*. The betweenness centrality of B1 (collapse), C2 (underground pipeline rupture) and C3 (road damage) events ranks the top three. Once such emergencies occur during subway construction and are not effectively controlled, they will cause serious negative effects.

(4) Comprehensive analysis of node importance

Based on the analytic hierarchy process, the comprehensive importance of network nodes is evaluated. According to the opinions of experts, the importance of the three evaluation indexes of degree centrality, closeness centrality and betweenness centrality of nodes is compared and analyzed, and the judgment matrix is constructed (Table 3). By combining the index weight value, the comprehensive importance of each node is calculated (Table 4). The results show that the top five events of comprehensive importance are collapse, underground pipeline rupture, road damage, impact on residents ‘lives and building damage.

**Table 3:** Comparison matrix of evaluation indexes.

Evaluating indicator	Degree of node	Closeness centrality	Betweenness centrality	Weight
**Degree of node**	1	1/2	1/2	0.2
**Closeness centrality**	2	1	1	0.4
**Betweenness centrality**	2	1	1	0.4

**Table 4:** Comprehensive evaluation of node importance in complex networks.

Node number	hazard event	degree	closeness centrality	betweenness centrality	node importance
B1	Collapse	1.8	0.184	35.2	37.184
C2	Underground pipeline rupture	1.8	0.212	27.36	29.372
C3	Road destruction	1.4	0.192	19.28	20.872
E5	Affect the lives of residents	1.2	0.176	15.2	16.576
C1	Damage of buildings	2	0.228	8.16	10.388
C4	Construction equipment damage	1.4	0.148	8.16	9.708
E4	Public opinion fermentation	0.4	0.24	8	8.64
E2	Loss of life	1.8	0.4	5.6	7.8
D10	Traffic tie-up	0.4	0.14	6.6	7.14
E1	Commercial closures	1.4	0.18	5.12	6.7

### 4.3. Identifying critical evolutionary paths for disasters based on edge indicators

The key evolution path analysis of the subway construction collapse is mainly to analyze the vulnerability of the edge, and the vulnerability is measured by the edge betweenness, average path length, and connectivity.

(1) Edge betweenness

The edge betweenness is a measure of the network intermediary, which refers to the proportion of the shortest path through a certain edge in the network. It can reflect the importance of a certain edge in the whole network [31], and the formula is [32]:


$$Be=\underset{i\neq j}{\sum }\frac{n_{ij}\left(e\right)}{n_{ij}}$$ (4)

where *Be* denotes the number of edge meshes of the edge *e*,$n_{ij}\left(e\right)$ is the number of times the shortest path between neighbouring nodes *i* and *j* in a complex network passes through a connected edge *e*. $n_{ij}$ denotes the sum of the number of shortest paths between node *i* and node *j.*

(2) Average path length

The average path length is the average distance between any two nodes and represents the degree of separation between nodes in a complex network, the larger the average path length, the more important this edge is [33]. The shorter the length of the shortest path of an edge, the greater the influence range of this connected edge, Eq [34]:


$$L=\frac{1}{n\left(n-1\right)}\sum \begin{array}{l} \left(i,j\in v\right) \end{array}d_{ij}$$ (5)

Where *L* is the average path length; *n* is the number of nodes;$d_{ij}$ is the distance from node *i* to node *j* in the network.

(3) Connectivity

Connectivity refers to the ratio of the number of nodes that can be connected to a starting node to the total number of nodes in a network. In a complex network, if the connectivity increases when an edge is removed, the surface will have a lower system risk in that network, equation is [35]:


$$H_{I}=\frac{N_{i}}{N}$$ (6)

Where *H*_*I*_ denotes the degree of connectivity, *N*_*i*_ is the number of nodes that the starting node *i* can connect to, and *N* is the total number of nodes.

(4) Vulnerability

The fragility of an edge is the degree of impact on the network if an edge is removed from the network. The higher the fragility, the more the edge affects the whole network, i.e., the more important it is, according to the formula [36]:


$$V_{e}=\frac{B_{e}L_{e}}{H_{e}}$$ (7)

Where $V_{e}$ is the fragility of edge *e*, $B_{e}$ is the number of edge mediators of edge *e*, $L_{e}$ is the average path length in the network after removing edge *e*, and $H_{e}$ refers to the connectivity in the network after removing edge *e*.

The vulnerability of 66 edges in the complex network of underground construction collapse disaster is brought into Eqs. (4)-(7), and the calculation results are arranged according to the highest to the lowest (Table 5) for the top ten ranked edge vulnerability.

**Table 5:** Edge vulnerability of complex network model of urban subway construction collapse disaster.

Start→Finish	Edge betweenness	Average path length	Connectivity	Vulnerability	Sort
B1 → C2	0.11	2.376	1	0.239	1
C3 → D10	0.07	2.424	1	0.172	2
B1 → C1	0.08	2.330	1	0.171	3
A2 → B1	0.07	2.267	0.962	0.158	4
A3 → B1	0.07	2.267	0.962	0.158	5
A4 → B1	0.07	2.267	0.962	0.158	6
E1 → E4	0.05	2.35	1	0.127	7
B1 → C3	0.05	2.35	1	0.121	8
E2 → E7	0.05	2.32	1	0.110	9
D10 → E5	0.05	2.37	1	0.109	10

Through the analysis of 66 key edges in the complex network of subway construction collapse disaster, it is concluded that the edges with higher vulnerability in the complex network are B1(collapse) → C2(underground pipeline rupture), C3(road damage) → D10(traffic paralysis), B1(collapse) → C1(building damage), A2(non-standard construction technology) → B1(collapse), A3(construction equipment failure) → B1(collapse). It can be concluded that if the propagation path of these edges is cut off, it will have a greater impact on the disaster network and prevent the further evolution of the disaster chain, so priority will be given to cutting off the edge that evolved into the rupture of the underground pipeline after the underground construction collapse, which will have a significant inhibition on the propagation of the disaster chain.

## 5. Analysis of disaster mitigation measures for underground construction collapse disaster chain breakage

According to the visual topology diagram of the complex network of urban subway construction collapse disaster, it can be seen that the occurrence of the accident is the result of the interaction of various factors. Based on the vulnerability analysis results of key nodes and key edges proposed in this paper, combined with accident lessons and related safety management methods, the countermeasures for chain breaking and disaster reduction are proposed.

(1) Comprehensively improve the safety efficiency of enterprises. Construction equipment failures and human-managed defects, which are at the forefront of vulnerability rankings, are closely related to corporate responsibility. Therefore, enterprises should increase capital investment in safety production, and improve the reliability and safety of construction equipment through reasonable planning of safety construction budget. Top managers of enterprises need to establish correct safety awareness, improve safety management system and strengthen safety education, actively advocate and supervise regular safety meetings, safety knowledge training and safety behavior assessment, promote the cultivation of enterprise safety culture, and ensure that the concept of safety construction is deeply rooted in the hearts of every employee.
(2) Strengthen on-site supervision and intelligent management. In order to prevent the construction collapse and underground pipeline rupture caused more serious impact. The supervisors need to improve the daily supervision intensity of the construction unit and increase the frequency of on-site inspections, so as to timely identify and investigate the illegal operations of the construction unit and prevent the construction personnel from carrying out potential safety risk behaviors. Promote the construction of smart construction sites, deploy a variety of monitoring facilities at the construction site, and use computers and mobile phone applications to achieve comprehensive and real-time dynamic management and safety monitoring of personnel, materials, machinery and environment.
(3) Optimize the comprehensive management of front-line construction personnel. Due to the non-standard technical operation of construction personnel, collapse accidents occur frequently. Scientifically allocate jobs, ensure that key positions are certified, strictly regulate construction operations, and implement reward and punishment mechanisms. The construction personnel are organized to study the construction organization design seriously, strictly follow the construction requirements, and put an end to the illegal operation behavior.

## 6. Conclusion

### 6.1. Generalize

In this paper, through the statistics of typical cases of subway construction collapse at home and abroad, with the help of complex network theory and disaster chain theory, the evolution process of collapse disaster is analyzed, the network model of subway construction collapse disaster chain is constructed, and the chain breaking disaster reduction strategy is systematically analyzed and discussed. The following conclusions are drawn:

(1) With the help of Gephi software, each disaster element in the urban underground construction collapse disaster chain is converted into a network node and numbered, with a total of 27 key nodes; according to the statistical analysis of urban underground construction collapse accidents, the evolution between the network nodes is weighted, and 66 key edges are obtained, which then draws a topological map of the complex network visualisation of urban underground construction collapse disaster.
(2) By calculating the degree, closeness centrality and betweenness centrality of nodes, the importance of network nodes is comprehensively analyzed. The top five events are collapse, underground pipeline rupture, road damage, affecting the lives of residents and building damage.
(3) The vulnerability of the network is analyzed by calculating the edge betweenness, average path length and connectivity, and the results show that the edges with high vulnerability are collapse →  underground pipeline rupture, road damage →  traffic paralysis, collapse →  building damage, so priority can be given to cutting off these edges with high vulnerability to destroy the entire topology of the network.
(4) Based on the analysis of key nodes and key edges in the complex network of underground construction collapse disasters, a number of measures to destroy key node events and cut off key propagation paths have been proposed to achieve the purpose of breaking the chain of disaster mitigation for the reference of the government departments in disaster prevention and mitigation work.

### 6.2. Limitations and future work

This study focuses on cutting off the chain reaction of collapse disasters during subway construction, and does not analyze the cost of risk control in depth. Therefore, in the future, based on this, according to the typical subway construction safety risk control scenarios in reality, this paper will analyze the risk control cost with the probability reasoning results under different scenarios, and promote the sustainable development of subway construction.

## Supporting information

S1 File Vulnerability. (XLSX)

S2 File Average path length. (XLSX)

S3 File Connectivity. (XLSX)

S4 File Edge betweenness. (XLSX)
